# Mobile plaque in the internal carotid artery: A case report and review

**DOI:** 10.4103/0972-2327.56320

**Published:** 2009

**Authors:** Dheeraj Khurana, Monica Saini, Judy Laldinpuii, N. Khandelwal, S. Prabhakar

**Affiliations:** Department of Neurology, Postgraduate Institute of Medical Education and Research, Chandigarh - 160 012, India; 1 Department of Radiodiagnosis, Postgraduate Institute of Medical Education and Research, Chandigarh - 160 012, India

**Keywords:** Carotid duplex, mobile plaque

## Abstract

A mobile plaque in the carotid artery is an uncommon entity, usually detected incidentally on a carotid duplex scan or angiography. It is associated with an indeterminate risk of an embolic stroke and should be managed on an emergent basis. We report here a case of a mobile plaque in the internal carotid artery that was detected serendipitously in a carotid duplex scan.

## Introduction

Plaque morphology on duplex ultrasound is an independent risk factor for ischemic stroke.[1] A mobile plaque in the carotid artery, an uncommon entity, was detected incidentally in a carotid duplex scan or during angiography.[2,3]

## Case Report

**Figure 1:**
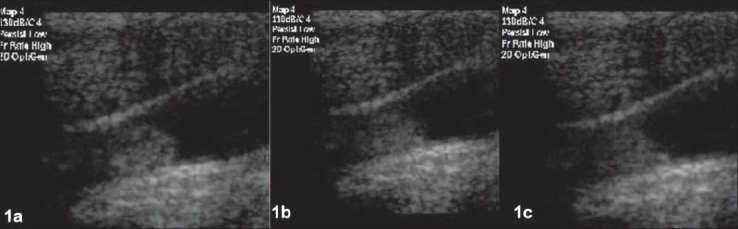
(a, b, c) B-mode carotid doppler (longitudinal) showing the mobile plaque in the proximal left ICA moving in sequence during systole

A 65 year-old diabetic lady presented at 6 hours of awakening from sleep with right-sided weakness and inability to speak and comprehend. Examination revealed right hemiparesis, right facial palsy, and global aphasia; there was no carotid bruit. Biochemistry showed a high fasting blood sugar level (260 mg%), raised serum creatinine level (1.7 mg%), a deranged lipid profile: cholesterol (T) = 237, LDL = 140, HDL = 48, triglycerides = 244. 2D echocardiography was unremarkable and cranial CT scan was normal at the time of admission although a repeat CT scan done 24 hours later showed a left middle cerebral artery (MCA) infarct. A carotid duplex study showed an intimal medial thickness (IMT) of 0.08 cm and a hyperechoic mobile plaque in the origin of the left internal carotid artery (ICA) [Figure 1, Video 1]. The patient was started on statins and heparin infusion. The patient refused an urgent endarterectomy and oral anticoagulation was continued. Over the next few days, there was significant recovery of power on the right side (MRC grade: 4+/5). Five days after the first stroke, she developed abrupt onset weakness of her right side associated with drowsiness. Cranial CT Scan showed an increase in the infarcted area in the left MCA territory. The carotid duplex scan showed a complete occlusion of the left ICA by a hyperechoic plaque [Figure 2a]. Digital Subtraction Angiography showed a complete left ICA occlusion [Figure 2b]. The patient was discharged in a stable condition.

**Figure 2a:**
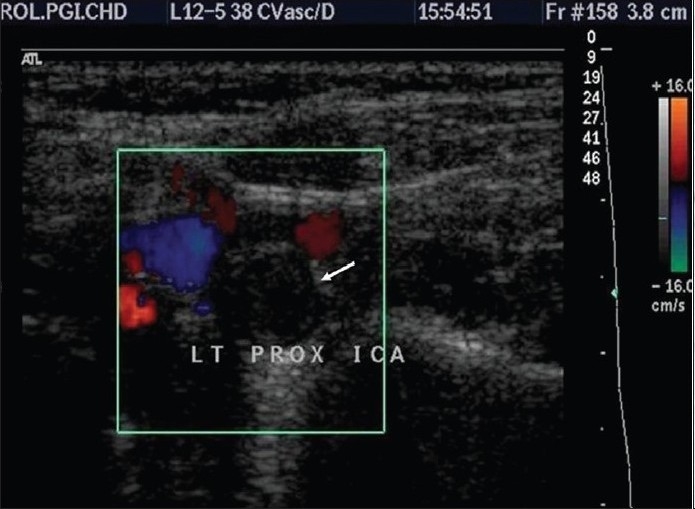
Color doppler (Transverse) showing occluded left ICA

**Figure 2b:**
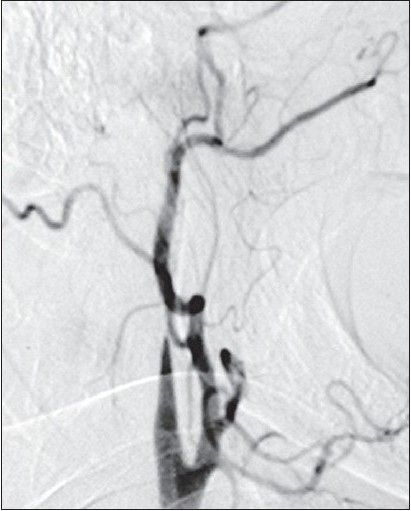
Digital Subtraction Angiography (DSA) showing left ICA occlusion

Video 1 Real time B mode and color duplex showing mobile plaque in the left ICA
See video on http://www.annalsofian.org

## Discussion

A mobile plaque in the carotid artery has an estimated prevalence of 1 in 2000[4] of asymptomatic individuals; its association with embolic cerebrovascular events is unknown.[4] Isolated case reports of mobile carotid plaques (MCP) in the past have demonstrated that they are a strong predictor of stroke recurrence.[5,6] MCPs have been reported both in the common carotid artery (CCA) and the ICA.[2–4] These may be either degenerated atherosclerotic flaps, intimal dissection flaps, or mobile thrombi which may be attached to underlying ruptured plaques. Degenerated and ruptured lesions expose a thrombogenic nidus and thrombi can then extend further into the lumen. MCPs display unique mobility, synchronous with the cardiac cycle,[7] which may lead to stress on the attaching pedicle of the mobile structure with resultant distal embolization.

Duplex ultrasound provides a sensitive and specific evaluation of plaque morphology and mobile structures in the vessel lumen. Previous case reports describing MCPs have included patients with underlying cardiac disease. A few asymptomatic patients, some of whom had a past history of CEA (Carotid Endarterectomy), have been incidentally detected with mobile plaques on follow-up duplex examination.[8] Our patient did not have any previous history of cardiac disease and after presentation, was detected to have an MCP on routine carotid duplex examination whereas her echocardiogram was normal.

Kimura and Uchino have described the characteristics of intravascular mobile structures detected by duplex ultrasound in five patients (three in the proximal ICA and two in the CCA). All their patients had cardioembolic stroke (four had atrial fibrillation and one had an acute myocardial infarction). In three of their patients, recanalization was seen on follow-up duplex scans, and in two patients, the mobile mass gradually changed to a immobile, hyperechoic structure.[7] Schalachetzki *et al*. described one patient of cardioembolic stroke with a mobile atheroma in the CCA. In patients with CCA MCPs and a cardiac source of emboli, the IMT thickness is usually unaffected.[9] Our patient had no obvious source of embolization. On duplex examination, the IMT was found to be increased, indicating that underlying atherosclerotic disease of the carotid vessels was the likely etiology. The mobile plaque in our patient went on to develop symptomatic complete occlusion of the ICA despite anticoagulation.

Cho *et al*. described a patient of acute stroke with a free-floating thrombus in the CCA with no cardioembolic source.[5] Stewart *et al*. reported a patient with a mobile lesion in the ICA who presented with only visual loss.[6] Combe *et al*. have reported six patients with free-floating clots diagnosed by arteriography in the ICA; three of their patients had an evolving stroke. In three patients, an underlying ulcerated plaque was seen, two had an atheromatous stenosis and one patient had arterial dissection.[10] Ko *et al*. described two patients with multiple cerebral infarcts caused by embolization from a free-floating thrombus at the bifurcation of the carotid.[11] Most patients with MCPs are thus neurologically unstable and at a high risk of recurrent stroke, which was well demonstrated in our case.

Recommendations for the management of MCPs include urgent carotid endarterectomy (CEA), embolectomy, or carotid angioplasty and stenting (CAS) in the case of previously endarterectomized carotid arteries. Anticoagulation has also been tried in a few cases. Szendro *et al*. treated two asymptomatic patients with MCPs with warfarin; both showed resolution without any neurological event.[8] Combe *et al*. managed six patients with MCPs in the ICA with intravenous heparin over two to five weeks. There was complete clot lysis in four patients, partial lysis in one patient, and one patient showed moderate extension of the clot.[10] However, carotid surgery was later performed in five of these patients for major carotid lesions and residual clots. Although traditional teaching mandates a six-week waiting period between stroke and carotid surgery, patients with MCPs who have suffered an acute event, are neurologically unstable with a high risk of recurrence and warrant treatment with urgent CEA. Chakhtoura *et al*. recommend stenting and anticoagulant medication in patients with an MCP and a past history of CEA.[12] Our patient presented with acute stroke, and as she refused CEA, she was managed with anticoagulation, despite which the mobile plaque progressed to complete ICA occlusion and manifested clinically as a recurrent stroke. This supports the contention that urgent CEA is mandatory in neurologically unstable patients in whom a mobile carotid plaque is the underlying etiology of stroke.

Mobile plaques may be detected serendipitously and may present with remarkable variability and are strong predictors of stroke recurrence.
