# Supplementary figures and images for: Mobile plaque in the internal carotid artery: A case report and review
Source: Ann Indian Acad Neurol. 2009 Jul-Sep;12(3):185–7. doi: 10.4103/0972-2327.56320 (PMC2824937; doi:10.4103/0972-2327.56320)

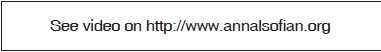

Supplement: Video 1 — Real time B mode and color duplex showing mobile plaque in the left ICA See video on http://www.annalsofian.org [file AIAN-12-185-g004.tif]
